# Restructuring the clinical curriculum at University Medical Center Göttingen: effects of distance teaching on students’ satisfaction and learning outcome

**DOI:** 10.3205/zma001397

**Published:** 2021-01-28

**Authors:** Theresa Seifert, Tim Becker, Amelie Friederike Büttcher, Nadine Herwig, Tobias Raupach

**Affiliations:** 1 Universitätsmedizin Göttingen, Bereich Medizindidaktik und Ausbildungsforschung, Göttingen, Germany; 2 Universitätsmedizin Göttingen, Klinik für Kardiologie und Pneumologie, Göttingen, Germany

**Keywords:** digital teaching, distance teaching, curriculum, learning outcome, evaluation

## Abstract

**Introduction:** In summer term 2020, the clinical phase of the undergraduate medical curriculum at University Medical Center Göttingen was restructured since distance teaching had to be used predominantly due to contact restrictions during the COVID-19 pandemic. This paper investigates the impact of restructuring the clinical curriculum on medical students’ satisfaction and learning outcomes.

**Methods: **In each cohort, the 13-week curriculum was divided into two parts: During the first 9 weeks, factual knowledge was imparted using distance teaching by means of a modified inverted classroom approach. This was followed by a 4-week period of adapted classroom teaching involving both real and virtual patients in order to train students’ practical skills. The evaluation of the 21 clinical modules comprised students’ satisfaction with distance teaching as well as students’ learning outcome. The latter was assessed by means of comparative self-assessment (CSA) gain and the results of the module exams, respectively. Data of summer term 2020 (= distance teaching, DT) were compared with respective data of winter term 2019/20 (= classroom teaching, CT) and analysed for differences and correlations.

**Results: **Response rates of evaluations were 51.3% in CT and 19.3% in DT. There was no significant difference between mean scores in module exams in CT and DT, respectively. However, CSA gain was significantly lower in DT (p=0.047) compared with CT. Further analyses revealed that CSA gain depended on the time point of data collection: CSA gain was lower the more time had passed since the end of a specific module. Moreover, we found positive correlations between CSA gain and students’ satisfaction with various aspects of distance teaching, particularly with “communication between teachers and students” (rho=0.674; p=0.002).

**Discussion and conclusions:** Although some limitations and confounding factors have to be taken into account (such as evaluation response rates, assessment time points, and proportion of familiar items in module exams), the following recommendations can be derived from our findings:

1. A valid assessment of students’ learning outcome by means of exam results requires that as few exam items as possible are familiar to the students.
2. CSA gain seems to be valid if assessment time points are standardised and not contaminated by students’ learning activities for other modules.
3. Good communication between teachers and students may contribute to increase students’ satisfaction with distance teaching.

## Zusammenfassung

**Einleitung: **Aufgrund der Kontaktbeschränkungen während der COVID-19-Pandemie musste die Lehre an der Universitätsmedizin Göttingen im Sommersemester 2020 auf vorwiegend digital-gestützte Formate umgestellt werden. Im vorliegenden Beitrag wird untersucht, inwieweit sich die Umstrukturierung des klinischen Curriculums auf den Lernerfolg der Medizinstudierenden auswirkt.

**Methoden: **Im Zuge der Umstrukturierung wurde in jeder Semesterkohorte ein 9-wöchiger Theorieblock gebildet, in dem die Lehrinhalte digitalisiert und mithilfe des Inverted-Classroom-Ansatzes vermittelt wurden. Hierauf folgte ein 4-wöchiger Praxisblock, in dem Unterricht in Präsenz mit realen und virtuellen Patient*innen stattfand. In der Evaluation wurden in allen 21 Modulen des klinischen Studienabschnitts verschiedene Daten erfasst: Neben der Zufriedenheit der Studierenden mit der digitalen Lehre wurde der studentische Lernerfolg einerseits anhand vergleichender Selbsteinschätzungen (VSE) und andererseits über Ergebnisse der Modulklausuren erhoben. Die Daten des digitalen Semesters (DS) wurden mit den entsprechenden Daten aus dem vorangegangenen Präsenzsemester (PS) verglichen und auf Unterschiede und Korrelationen geprüft.

**Ergebnisse: **Der Rücklauf der Evaluationen betrug im PS 51,3% und im DS 19,3%. Zwischen den gemittelten Klausurleistungen im PS und DS gab es keinen signifikanten Unterschied. Allerdings nahm der VSE-Lernerfolg im DS signifikant ab (p=0,047). Eine weiterführende Analyse zeigte, dass der VSE-Lernerfolg im DS umso geringer eingeschätzt wurde, je mehr Zeit zwischen Modulende und Evaluation lag. Der VSE-Lernerfolg korrelierte mit der studentischen Zufriedenheit mit verschiedenen Aspekten der digitalen Lehre, insbesondere der Kommunikation zwischen Lehrenden und Studierenden (rho=0,674; p=0,002).

**Diskussion und Schlussfolgerungen: **Unter Berücksichtigung verschiedener Limitationen und Störfaktoren – wie z.B. Evaluationsrücklaufquoten, Evaluationszeitpunkte, Anteil von Altfragen in den Modulklausuren – lassen sich folgende Empfehlungen ableiten:

1. Eine valide Messung des Lernerfolgs über Klausurergebnisse setzt voraus, dass möglichst wenige bereits bekannte Fragen verwendet werden.
2. Der VSE-Lernerfolg kann als valide angesehen werden, wenn die Evaluationszeitpunkte standardisiert sind und Überlappungen mit anderen Lernaktivitäten vermieden werden.
3. Im Sinne studentischer Zufriedenheit mit digitaler Lehre sollte insbesondere auf eine gute Kommunikation zwischen Studierenden und Lehrenden geachtet werden.

## Introduction

At University Medical Center Göttingen, the clinical phase of the undergraduate medical curriculum is made up of 21 interdisciplinary modules, each lasting 2 to 7 weeks and comprising both factual knowledge teaching and practical skills training. In summer term 2020, the clinical curriculum was restructured since distance teaching had to be used predominantly due to contact restrictions during the COVID-19 pandemic. The aim of this paper is to outline the restructuring of our clinical curriculum and to investigate and discuss the impact of this process on medical students’ satisfaction and learning outcome.

## Methods

**Figure 1:**
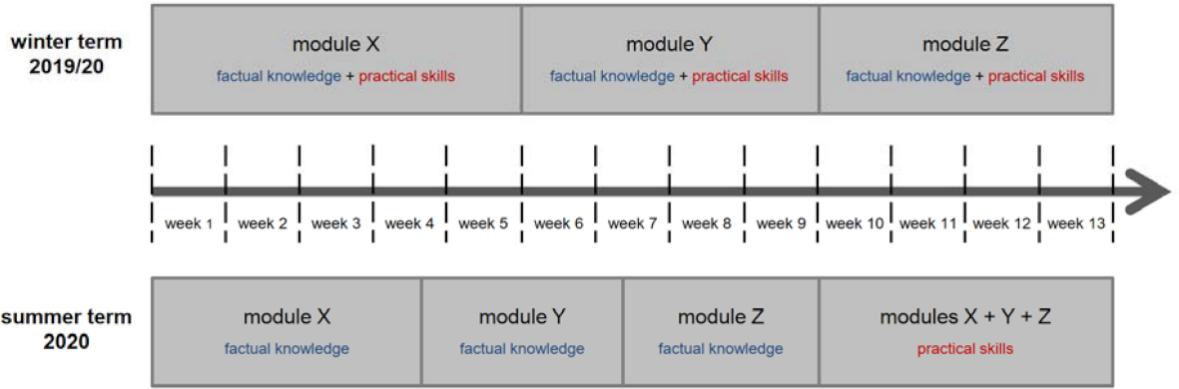
Scheme of the separation of factual knowledge teaching and practical skills training within one cohort in summer term 2020 compared to winter term 2019/20.

In the course of restructuring, the 13-week curriculum of each cohort was divided into a 9-week period of distance teaching (DT) and a subsequent 4-week period of modified classroom teaching (CT; see figure 1 (Fig. 1)).

During the DT period, students were provided with access to digital teaching material (such as video lectures) which had been produced by teachers of the respective modules. According to the inverted classroom approach [1], [2], students gained factual knowledge by watching the videos and, afterwards, applied and deepened their knowledge by solving case-based clinical problems. However, students’ elaboration did not take place in the classroom (as is the case in the classical concept) but by submitting their reports to their teachers and getting feedback via e-mail. In addition to these asynchronous teaching formats, synchronous videoconferencing was provided as well in order to enable real-time interaction between students and teachers.

Following the 9-week distance teaching period, a 4-week period of classroom teaching (which was adapted to the actual contact restrictions) was conducted across all modules of the respective cohort (see figure 1 (Fig. 1)). During this phase, practical skills training involving both real and virtual patients [3] (e.g. by playing a serious game [4]) was offered.

As part of the evaluation of summer term 2020, various data were collected and aggregated in each of the 21 clinical modules. Students’ satisfaction with various aspects of distance teaching was assessed by means of an online questionnaire comprising five items scored on six-point scales. Students’ learning outcome was derived from both comparative self-assessments (CSA) [5] and the results of the written module exams. Due to the fact that the evaluation had to be adapted to the new teaching formats rather quickly, the assessment time points varied across modules: only a few modules were evaluated immediately at the end while others were evaluated with a delay of several weeks.

Data regarding students’ learning outcome in summer term 2020 (DT) were compared with respective data of winter term 2019/20 (CT). Differences were analysed using the Mann-Whitney U test. Additionally, Spearman’s rank correlation was calculated for several correlational analyses. Inferential statistical tests were performed with SPSS Statistics 26.

## Results

In summer term 2020, a total of 928 students in six cohorts were enrolled in the 21 clinical modules. The median response rate of evaluations was 19.3%. In contrast, it had been 51.3% in the previous winter term 2019/20.

**Figure 2:**
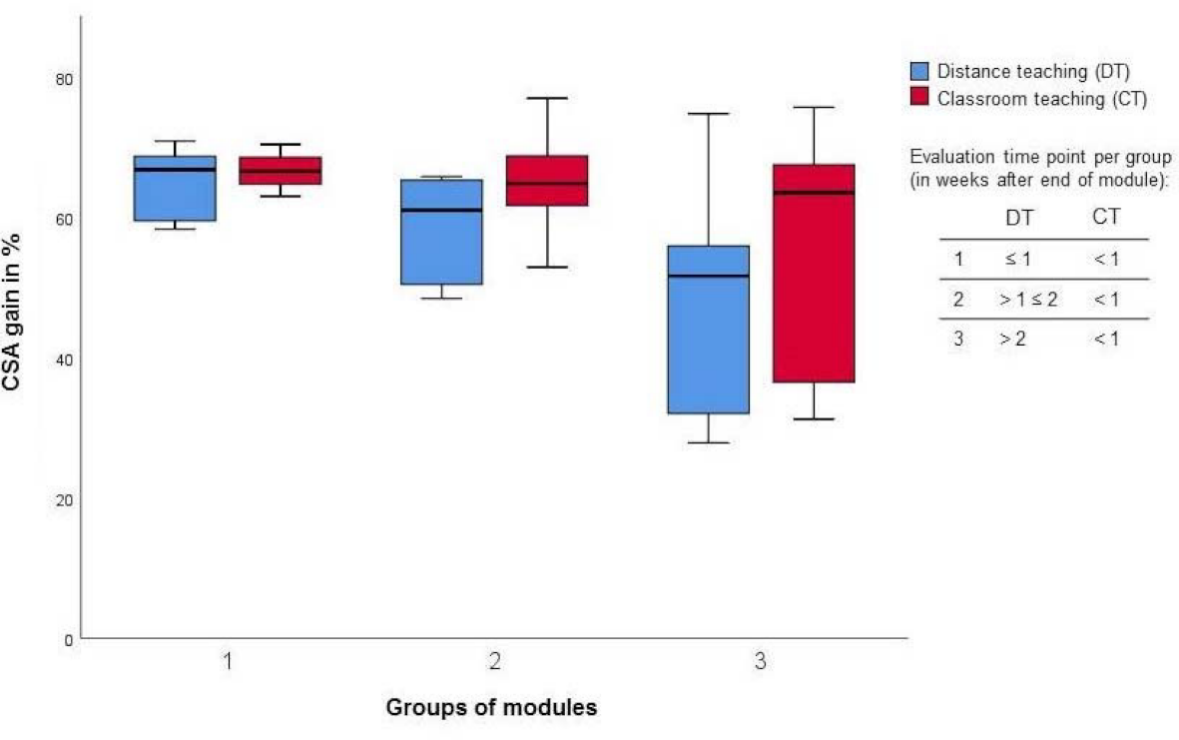
Comparison of mean CSA gain in CT and DT depending on evaluation time point (weeks after end of module).

Regarding the comparison of students’ learning outcome, there was no significant difference between mean scores in module exams in CT and DT, respectively. However, learning outcome expressed as CSA gain (which was averaged across a total of 304 specific learning objectives) decreased significantly in DT (p=0.047) when compared with CT. A more detailed analysis separating cognitive and practical aspects showed no significant difference between CT and DT in terms of cognitive learning objectives (*Mdn_CT_*=65.0%, IQR 10.8% vs. *Mdn_DT_*=59.1%, IQR 11.2%, p=0.328) but a significant decrease regarding practical learning objectives in DT compared with CT (*Mdn_CT_*=62.7%, IQR 17.6% vs. *Mdn_DT_*=55.4%, IQR 19.4%, p=0.036). Further analyses revealed that CSA gain depended on the time of data collection: It was lower the more time had passed since the end of module (see figure 2 (Fig. 2)). In contrast, when assessment time points were standardised across the modules (as in CT), no significant difference in students’ CSA gain was detected.

Furthermore, CSA gain correlated significantly with students’ satisfaction with various aspects of distance teaching: the strongest correlation was found between the CSA learning outcome (aggregated at module level) and students’ ratings of the item “The teacher(s) responded well to questions and suggestions” (rho=0.674; p=0.002).

## Discussion and conclusions

Our study aimed at investigating the impact of restructuring the clinical curriculum (i.e. the switch to distance teaching) on medical students’ satisfaction and learning outcome. The comparison between CT and DT showed no significant difference regarding mean scores in module exams. However, students’ CSA gain was significantly lower in DT, particularly in terms of practical learning objectives. This finding had been expected due to the required suspension of several practical teaching formats during DT.

One limitation of both learning outcome parameters is that they are potentially biased by some confounding factors: Our finding that mean scores in the module exams did not differ between CT and DT might be explained by the fact that some modules used a high proportion (up to 100%) of similar or even identical exam items than in the previous term (CT). This phenomenon of familiar exam items is relevant for traditional classroom teaching as well but it first became apparent within the scope of our detailed analyses of DT. Thus, our findings also provide further approaches for revising exams at our faculty.

Another confounding factor is that students’ CSA gain apparently varies depending on data collection time points: We found lower CSA gain if data were collected with a longer lag after the end of a module. In contrast, this trend had not been observed in CT when each clinical module had been evaluated immediately at its end. One probable reason for such findings might be a decrease in knowledge retention over time. Therefore, in order to be able to compare students’ learning outcome across modules, evaluation time points should be standardised for all modules.

Finally, the interpretation of CSA gain in DT is also limited due to the lower response rate of evaluations compared to CT. This is why it is not possible to draw firm conclusions on which parts of the new curriculum should be retained.

Summarising the above mentioned aspects, the following recommendations for both distance teaching and classroom teaching can be derived: Results of module exams are valid indicators of students’ learning outcome only if exams do not predominantly consist of items that are familiar to the students. CSA gain might validly reflect students’ actual learning outcome if assessment time points are standardised and are not contaminated by students’ learning activities for other modules. Lastly, good communication between teachers and students may contribute to increase students’ satisfaction with distance teaching.

## Authorship

Theresa Seifert and Tim Becker share the first authorship.

## Competing interests

The authors declare that they have no competing interests.
